# Effect of Acupuncture on Intraocular Pressure in Glaucoma Patients: A Single-Blinded, Randomized, Controlled Trial

**DOI:** 10.1155/2020/7208081

**Published:** 2020-04-28

**Authors:** Shu-Yuan Chen, Feng-Shuen Yieh, Wen-Ling Liao, Tsai-Chung Li, Ching-Liang Hsieh

**Affiliations:** ^1^Graduate Institute of Chinese Medicine, College of Chinese Medicine, China Medical University, Taichung 40402, Taiwan; ^2^Dashe Shun Ming Eye Clinic, Kaohsiung 815, Taiwan; ^3^Graduate Institute of Integrated Medicine, College of Chinese Medicine, China Medical University, Taichung 40402, Taiwan; ^4^Center for Personalized Medicine, China Medical University Hospital, Taichung, 40402, Taiwan; ^5^Graduate Institute of Biostatistics, College of Public Health, China Medical University, Taichung 40402, Taiwan; ^6^Department of Healthcare Administration, College of Health Science, Asia University, Taichung 413, Taiwan; ^7^Chinese Medicine Research Center, China Medical University, Taichung 40402, Taiwan; ^8^Graduate Institute of Acupuncture Science, College of Chinese Medicine, China Medical University, Taichung 40402, Taiwan; ^9^Department of Chinese Medicine, China Medical University Hospital, Taichung 40447, Taiwan

## Abstract

Glaucoma is characterized by the degeneration of retinal ganglion cells that cause progressive optic neuropathy, finally resulting in changes to the optic nerve head. Lowering intraocular pressure (IOP) is the only method proven for treating glaucoma. Several studies have discovered that acupuncture can reduce IOP and also increase ocular perfusion and ocular blood flow. Therefore, the present study investigated the effect of acupuncture on IOP in glaucoma patients. We conducted a single-blinded, randomized, controlled trial involving 45 glaucoma patients. The results indicated that the difference between the IOP 60 min after the intervention and IOP immediately before the intervention was greater in the acupuncture group (AG) and electroacupuncture group (EG) than in the sham group (SG) for all four of the interventions performed and in both eyes (all *p* < 0.05). The IOP difference between immediately before the first intervention and after finishing the final intervention was also greater in the AG and EG than in the SG in both eyes (all *p* < 0.05). In conclusion, IOP was reduced at 60 min after acupuncture or electroacupuncture was performed at BL1 and EX-HN7. Additionally, IOP was reduced after finishing four acupuncture or electroacupuncture sessions. Therefore, our results suggest that acupuncture and electroacupuncture are beneficial for lowering IOP in glaucoma patients. This trial is registered with NCT04157530.

## 1. Introduction

Glaucoma is a leading cause of blindness after cataract; it is an optic neuropathy with a specific structural finding in the optic disc and causing a specific functional deficit in automated visual field examinations [1]. According to its pathophysiology and treatment, glaucoma is classified into two main types: open angle and closed angle [2]. The global prevalence of glaucoma is 3.54% among those aged between 40 and 80 years. The prevalence of primary open-angle glaucoma is the highest in Africa at 4.20%, whereas that of primary closed-angle glaucoma is the highest in Asia at 1.09%. The number of people aged 40–80 years with glaucoma is expected to increase from 64.3 to 76.0 million in 2020 and 111.8 million in 2040 [3]. The crude prevalence of all glaucoma is 3.8%, primary open-angle glaucoma is 2.1%, and primary closed-angle glaucoma is 1.5% in the population of southern China [4].

Glaucoma is characterized by the degeneration of retinal ganglion cells, causing progressive optic neuropathy and finally resulting in changes to the optic nerve head [2]. The main clinical manifestations of glaucoma are visual field defects and irreversible blindness, but a lack of clinical symptoms is possible in the early stage. Both old age and high intraocular pressure (IOP) are risk factors for glaucoma development [5]. Decreased ocular perfusion and ocular blood flow may also play roles in the development of open-angle glaucoma [6, 7]. Furthermore, impairment of ocular vascular autoregulation has been reported in patients with normal-tension glaucoma [8]. Lowering IOP is the only method proven to treat glaucoma, and IOP can be lowered through methods such as ocular hypotensive drops, laser trabeculoplasty, and surgery [2, 9]. Acupuncture treatment resulted in significantly lower IOP than sham acupuncture treatment in a 4-week clinical trial involving glaucoma patients [10]. Acupuncture performed at eye-specific acupoints can increase ocular blood flow in patients with primary open-angle glaucoma [11].

Acupoints Jingming (BL1) and Qiuhou (EX-HN7) are located in the periorbital region, and according to traditional Chinese medicine theory, BL1 can communicate yin and yang and nourish and clear eyes, whereas Qiuhou (EX-HN7) can transport qi, activate the blood, and achieve free-flowing meridians and clear eyes. The present study investigated the effect of acupuncture at BL1 and EX-HN7 on IOP in glaucoma patients. We designed a single-blinded, randomized, controlled clinical trial.

## 2. Materials and Methods

### 2.1. Subjects

The study participants were patients with glaucoma that had been diagnosed by an ophthalmologist from Dashe Shun Ming Eye Clinic, Kaohsiung, Taiwan. Acupuncture was performed at the Chi-Sin Chinese Medicine Clinic between March and October 2018 by a Chinese medicine doctor with at least 10 years of acupuncture practice experience. The research protocol was reviewed and approved by the Research Ethics Committee of China Medical University Hospital (approval number: CMUH106-REC2-161) and ClinicalTrials.gov (trial registration number: NCT04157530). The purpose of the study and all procedures of the trial were explained to the participants, and informed consent was obtained prior to the trial. The inclusion criteria were as follows: (1) glaucoma diagnosis at least 3 months previously; (2) use of one or no intraocular hypotensive drugs; (3) age ≥20 years; (4) female or male; and (5) clear consciousness that enabled the participant to sign the informed consent form and cooperate with the trial procedure. The exclusion criteria were (1) comorbidity with other chronic diseases, such as hypertension and diabetes, and taking multiple drugs; (2) laser surgery for either glaucoma or myopia; (3) pregnancy or lactation; (4) intolerance to acupuncture treatment; (5) allergy to acupuncture needles; and (6) refusal to sign the informed consent form.

### 2.2. Study Design and Sample Size Calculation

The present study was designed as a single-blinded, randomized, placebo-controlled, three-armed trial. Each participant was treated with sham acupuncture, acupuncture, or electroacupuncture (EA) twice weekly for 2 consecutive weeks (four interventions), each intervention was 20 min. IOP was evaluated at four time points for each treatment. Assuming an effect size of 0.50 and a type I error (*α*) of 0.05, 39 people (13 in each group) were required to achieve a statistical power of 90%. Assuming a dropout rate of 5%, 45 people (15 in each group) were needed.

### 2.3. Randomization and Grouping

The participants were divided into three groups through random number table allocation after obtaining their signed informed consent: (1) the sham group (SG), to which the seeds of Wang Bu Liu Xing, a Chinese herb, were applied to the skin surface at BL1 and EX-HN7 for 20 min at a time; (2) the acupuncture group (AG), to which an acupuncture needle (34 no., MCN1294000, Maanshan Bond Medical Instruments Co., Ltd., Anhui Province, China) with a spherical needle head was inserted into BL1 and EX-HN7 with Der-qi; and (3) the electroacupuncture group (EG), to which needles were inserted similarly as in the AG group, but the needles were then connected to an EA machine after Der-qi, with the frequency of stimulus being 6 Hz and the intensity of stimulus enough to produce a minimal visible muscle twitch.

### 2.4. Assessment

Four stages of IOP assessments were performed for all the participants. The first IOP assessment (V1) was conducted before the sham, acupuncture, or EA intervention; the second assessment (V2) was conducted immediately after the intervention; the third assessment (V3) was conducted 30 min after the intervention; and the fourth assessment (V4) was conducted 60 min after the intervention. Quality of life was assessed using the World Health Organization Quality of Life brief (WHO Quality of Life-BREF), Taiwanese version; the scale was administered before the intervention and after the fourth intervention.

The primary outcome was IOP, and the secondary outcome was the change in the score of WHO Quality of Life-BREF, Taiwanese version.

### 2.5. Acupoints

**Figure 1:**
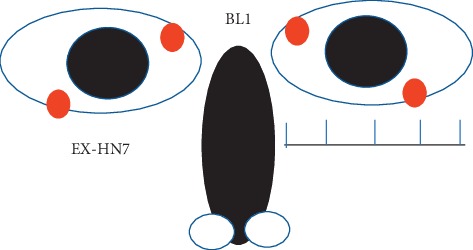
Location of Jingming (BL1) and Qiuhou (EX-HN7). BL1 is located in the depression between the superomedial parts and inner wall of the orbit. EX-HN7 is located at the junction between the outer 1/4 and medial 3/4 of the inferior margin of the orbit when the subject is siting up and looking upwards.

BL1 is located in the depression between superomedial parts and inner wall of the orbit. EX-HN7 is located at the junction between the outer 1/4 and medial 3/4 of the inferior margin of the orbit when the subject is in a sitting position and looking upwards (Figure 1).

### 2.6. IOP Measurement

A Canon fully automatic tonometer (MJP0295100, Canon Components Inc. Saitama, Japan) was used by a well-trained individual to measure IOP. In the present study, the average of three measurements was used. The IOP was measured from 9:00 a.m. to 11:30 a.m. to prevent the effects of diurnal intraocular pressure changes.

The visual field index (VFI) represented as % and mean defect (MD) represented as dB were measured by using a Humphrey Field Analyzer II-i (Carl Zeiss Meditec, Inc., USA) in the Dashe Shun Ming Eye Clinic (Kaohsiung, Taiwan) in some case.

The checklist of Consolidated Standards of Reporting Trials [12] was completed. The intervention was recorded in accordance with the Standards for Reporting Intervention in Clinical Trials of Acupuncture, which are summarized in Table 1 [13].

### 2.7. Statistical Analysis

Categorical data were displayed in numbers and percentages, whereas continuous variables were given as means ± standard deviations. Differences in continuous variables were tested using the analysis of variance or Kruskal–Wallis test and differences in categorical variables were tested using the chi-square test or Fisher exact test among groups. The repeated measures analysis of variance was used to compare the IOP differences between the four time points (V1–V4) for four interventions among different groups. A *p* value of less than 0.05 was considered statistically significant. All statistical analyses were performed using SAS 9.4 (SAS Institute, Cary, NC, USA) and SPSS Statistics 24 (IBM Corporation, Somers, NY, USA).

## 3. Results

**Figure 2:**
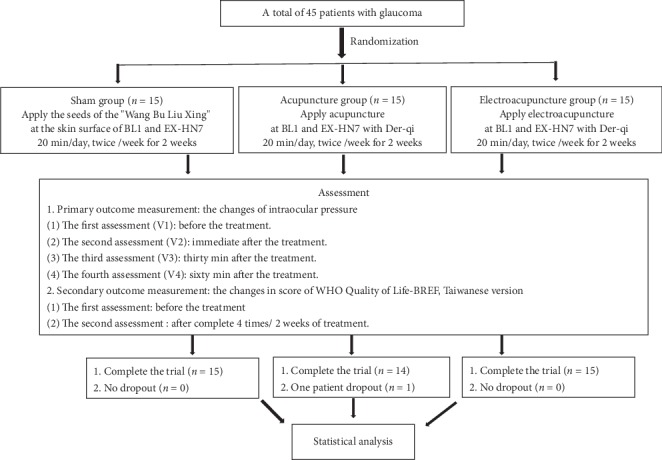
Flowchart of the trial.

A total of 44 glaucoma patients completed the trial: 15 patients in the SG, 14 in the AG, and 15 patients in the EG. One patient withdrew because he/she was afraid of the air that was suddenly ejected from the IOP measurement device (Figure 2).

The basic characteristics of the participants, their sex, age, height, body weight, blood pressure (systolic and diastolic blood pressure), IOP (right and left eyes), and latanoprost use for lowering IOP, showed no significant differences between the SG, AG, and EG (all *p* > 0.05; Table 2).

No bleeding or ecchymosis occurred at the acupuncture sites or in the orbital region in any patient.

### 3.1. Effect of Acupuncture on IOP in Glaucoma Patients

**Figure 3:**
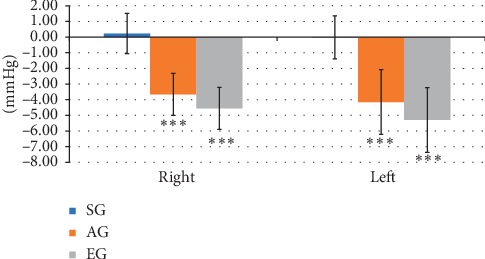
Effect of acupuncture and EA at BL1 and HX-EN7. The IOP difference between V1 in the first intervention and V2–V4 in the fourth intervention was greater in the AG and EG than in the SG. ^*∗∗∗*^*p* < 0.001 compared with the SG.

In the right eye, the V1 IOP showed no significant differences between the SG, AG, and EG for all four interventions (all *p* > 0.05; Table 3). The IOP differences between the four time points (V1–V4) for the four interventions were compared. The IOP differences between V4 and V1 were significantly greater in the AG and EG than in the SG in the first intervention (both *p*=0.001; Table 3). The IOP differences between V3 and V1 and between V4 and V1 were significantly greater in the AG and EG than in the SG in the second and third interventions (all *p* < 0.001; Table 3). Additionally, the IOP differences between V4 and V1 were greater in the AG and EG than in the SG in the fourth intervention (both *p*=0.002; Table 3). The difference in IOP between V2 and V1 was greater in the EG than in the SG or AG in the fourth intervention (both *p* < 0.001; Table 3). Lastly, the IOP difference between V1 in the first intervention and V2–V4 in the fourth intervention was compared and discovered to be greater in the AG and EG than in the SG (*p* < 0.001; Figure 3).

In the left eye, the V1 IOP showed no significant differences between the SG, AG, and EG in the second, third, and fourth interventions (all *p* > 0.05; Table 4), but the V1 IOP was greater in the EG than in the SG in the first intervention (*p*=0.03; Table 4). The IOP differences between V3 and V1 and between V4 and V1 were greater in the AG and EG than in the SG in the first intervention (*p*=0.01 for V3 vsV1; *p*=0.001 for V4 vs V1 for both group comparisons; Table 4). The IOP difference between V3 and V1 and between V4 and V1 was greater in the AG and EG than in the SG in the second, third, and fourth interventions (all *p* < 0.001; Table 4). Furthermore, the IOP difference between V2 and V1 was greater in the EG than in the SG in the third intervention (*p*=0.007; Table 4) and fourth intervention (*p*=0.004; Table 4). Finally, the IOP differences between V1 in the first intervention and V2–V4 in the fourth intervention were greater in the AG and EG than in the SG (*p* < 0.001; Figure 3).

### 3.2. Effect of Acupuncture on Visual Field in Glaucoma Patients

**Figure 4:**
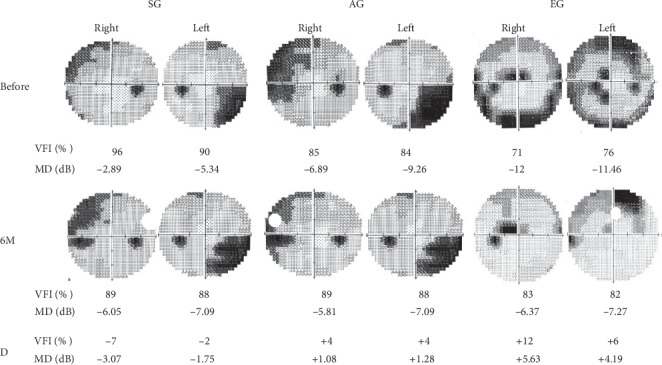
Effect of acupuncture and EA at BL1 and HX-EN7 on visual field. The visual field index (VFI) reduced 7% in the right eye (Right) and 2% in the left eye (Left); the mean defect (MD) reduced 3.07 dB in the right eye and 1.75 dB in the left eye in the sham group (SG). The VFI increased 4% in the right eye and left eye; the MD increased 1.08 dB in the right eye and 1.28 dB in the left eye in the acupuncture group (AG). The VFI increased 12% in the right eye and 6% in the left eye; the MD increased 5.63 dB in the right eye and 4.19 dB in the left eye in the electroacupuncture group (EG). Before, before the intervention of acupuncture or electroacupuncture; 6 M, 6 months after the intervention; D, the difference between B and 6 M.

There were three glaucoma patients, one in each group, and their visual field and MD were measured 6 months after acupuncture or EA intervention. The VFI reduced 7% in the right eye and 2% in the left eye; the MD reduced 3.07 dB in the right eye and 1.75 dB in the left eye compared to before the intervention in the SG. The VFI increased 4% in the right eye and in the left eye; the MD increased 1.08 dB in the right eye and 1.28 dB in the left eye compared to before the intervention in the AG. The VFI increased 12% in the right eye and 6% in the left eye; the MD increased 5.63 dB in the right eye and 4.19 dB in the left eye compared to before the intervention in the EG (Figure 4).

### 3.3. Effect of Acupuncture on the WHOQOL-BREF (Taiwanese Version) Score in Glaucoma Patients

The WHOQOL-BREF (Taiwanese version) assesses overall quality of life, overall health satisfaction, the physiological domain, the psychological domain, social relationships, the social relationship domain (Tw), environment, and the environmental domain (Tw). For this scale, scores obtained before any of the interventions and after any of the interventions did not show significant differences (all *p* > 0.05; Table 5).

## 4. Discussion

The results of the present study indicated that the IOP difference between V4 and V1 was greater in the AG and EG than in the SG for all four interventions and in both eyes. The difference between the V1 IOP before the first intervention and the V2–V4 IOP after the fourth intervention was significantly greater in the AG and EG than in the SG, but the difference between the AG and EG was not significant. In addition, the sex, age, height, body weight, blood pressure, IOP (both eyes), and latanoprost use showed no significant differences between the SG, AG, and EG. Therefore, these results demonstrate that acupuncture or EA at BL1 and HX-EN7 lowers IOP in glaucoma patients. The pathogenesis of glaucoma is increased IOP and ischemia of the optic nerve head, influencing the retinal ganglion axon. An ocular perfusion pressure below the lower limit of autoregulation or failure of neurovascular coupling results in secondary insults [7]. Abnormal ocular blood flow may play a crucial role in patients with normal-tension glaucoma [8]. Reduction in ocular perfusion pressure and ocular blood flow is also a risk factor in glaucoma patients [6]. Acupuncture improved the retrobulbar circulation and IOP of patients with open-angle glaucoma in a study using color Doppler imaging [14]. Pulsatile ocular blood flow was significantly increased from 5.6 to 6.7 *μ*L/min after eye-specific acupuncture treatment in a randomized clinical trial involving patients with primary open-angle glaucoma [11]. Taken together, these findings demonstrate that IOP, ocular perfusion, and ocular blood flow play critical roles in the development of glaucoma, and acupuncture has beneficial effects on IOP, ocular perfusion, and ocular blood flow in glaucoma patients, although the effects of acupuncture are short term. In addition, the mechanism through which acupuncture lowers IOP and increases ocular perfusion and blood flow remains unclear, and further research is required.

Our results also indicated that IOP was greater at V2 (immediately after the intervention) than at V1 (immediately before the intervention), suggesting a stress response immediately after sham, acupuncture, or EA treatment, especially for EA; however, this stress response had disappeared 30 min after the intervention. Our results showed good agreement with those of another study discovering that acupuncture temporarily increased IOP [15].

Although our results also indicated that the VFI and MD increased 6 months after acupuncture and EA interventions in the AG and EG, but reduced in the SG, only three cases could not be concluded, and future studies are needed to increase the number of cases.

Our results also indicated that the changes in all component scores and the total scores for the WHOQOL-BREF (Taiwanese version) showed no significant differences between the groups, suggesting that to investigate changes in quality of life, a longer investigation may be required. The intervention period in our study was only 2 weeks.

As for the duration of acupuncture or EA, and when is the best time for acupuncture or EA intervention to reduce intraocular pressure. According to the results of the present study (Tables 3 and 4), we found that the IOP of V1 (baseline) from the first to the fourth acupuncture or EA intervention both the right eye and left eye were gradually decreased in the AG and EG, but not in the SG. In addition, the IOP difference between V1 in the first intervention and V2–V4 in the fourth intervention was greater in the AG and EG than in the SG. The acupuncture or EA intervention was two times per week for two weeks continuously (four interventions) in the present study. Taken together, suggesting that acupuncture or EA twice weekly for 2 consecutive weeks (four interventions) can gradually reduce IOP in patients with glaucoma.

No bleeding, ecchymosis, or side effects such as shock occurred after acupuncture or EA was applied to BL1 and HX-EN7 around both eyes in this study. This suggests that applying acupuncture or EA to BL1 and HX-EN7 for a 2-week glaucoma treatment is safe in lowering intraocular pressure, but longer-term observation is required in the future. Taken together, we considered that acupuncture or EA is a better method in lowering IOP than medical therapies, such as latanoprost as one of the prostaglandin analogues because prostaglandin analogues may induce ocular side effects including blurred vision, dry eye, and hyperemia [9].

This study had some limitations: (1) the sample was small, the intervention period was short, and there was no long-term follow-up; therefore, larger samples, longer interventions, and follow-up are required in future research. (2) This was a single-blinded, randomized, controlled study; a double-blinded, randomized, controlled study should be designed in the future. (3) This study only assessed the effects of acupuncture or EA on IOP; how they affect visual acuity and the visual field should be determined in future studies. (4) Finally, this study did not differentiate between primary and secondary or open-angle and closed-angle glaucoma; classification of the glaucoma type is required in future research.

In conclusion, IOP was reduced 60 min after acupuncture or EA at BL1 and HX-EN7, and IOP was also reduced after finishing four acupuncture or EA sessions, suggesting that acupuncture and EA are beneficial for lowering IOP in glaucoma patients. In addition, acupuncture and EA at BL1 and HX-EN7 were discovered to be safe for 2 weeks.

## Figures and Tables

**Table 1:** Standards for reporting intervention in clinical trials of acupuncture (2010).

*Acupoint rationale*
(1) Meridian theory in the principles of traditional Chinese medicine
(2) Bilateral Jingming (BL1) and Qiuhou (EX-HN7)
Details of needling
(1) Apply the seeds of the “Wang Bu Liu Xing” that is one of the Chinese herbs to the both sides of the skin surface of BL1 and EX-HN7
(2) Acupuncture needles inserted into both BL1 and EX-HN7 acupoints
(3) Electroacupuncture apply to both BL1 and EX-HN7
*Treatment regimen*
(1) The seeds of the “Wang Bu Liu Xing,” acupuncture and electroacupuncture intervention 20 minutes at a time, twice a week for two consecutive weeks
(2) The frequencies of stimulus were 6 Hz; the intensity of stimulus was minimal visible muscle twitch in the electroacupuncture stimulation
*Comparator interventions*
(1) No herbs, moxibustion, cupping, massage, exercise, dietary advice, or lifestyle modification
*Practitioner background*
(1) The licensed traditional Chinese medicine doctor with over 540 h of acupuncture training and at least 10 years acupuncture clinical practice
*Control intervention*
(1) The seeds of the “Wand Bu Liu Xing” on the both sides of the skin surface of BL1 and EX-HN7

**Table 2:** Basic characteristics of glaucoma patients.

	SG (*n* = 15)	AG (*n* = 14)	EG (*n* = 15)	*p* value
Gender				0.98
Male	6	6	6	
Female	9	8	9	
Age	55.1 ± 11.9	43.6 ± 10.5	45.4 ± 10.5	0.23
Body length (cm)	163.9 ± 7.5	164.7 ± 6.8	166.4 ± 8.40	0.65
Body weight (kg)	62.4 ± 12.1	63.2 ± 11.1	66.3 ± 12.6	0.65
BP (mmHg)				
Systolic	112.5 ± 11.9	116.5 ± 10.5	115.3 ± 14.5	0.75
Diastolic	68.9 ± 5.50	71.5 ± 3.60	69.4 ± 5.80	0.86
IOP (mmHg)				
Right eye	16.95 ± 5.34	18.37 ± 3.91	19.77 ± 3.44	0.10
Left eye	16.88 ± 3.98	19.34 ± 3.91	20.66 ± 3.44	0.12
Latanoprost use	13	12	13	1.00

Data presented as mean ± standard deviation except for sex. SG, sham group; AG, acupuncture group; EG, electroacupuncture group; BP, blood pressure; systolic, systolic blood pressure; diastolic, diastolic blood pressure; IOP, intraocular pressure. Differences in continuous variables were tested using the analysis of variance or Kruskal–Wallis test; differences in categorical variables were tested using the chi-square test or Fisher exact test.

**Table 3:** Effect of acupuncture on right eye intraocular pressure in glaucoma patients (mmHg).

	SG (*n* = 15)	AG (*n* = 15)	EG (*n* = 15)	*p* value
First intervention				
V1	16.95 ± 5.34	18.73 ± 3.91	19.77 ± 3.44	0.21
V2	18.80 ± 5.73	20.32 ± 3.86	21.71 ± 3.29	0.21
V3	17.03 ± 5.43	17.66 ± 3.71	19.37 ± 3.24	0.31
V4	17.55 ± 5.70	17.69 ± 3.81	18.53 ± 3.50	0.81
V2-V1	1.85 ± 1.50	1.59 ± 1.20	1.94 ± 0.99	0.74
V3–V1	0.09 ± 1.76	−1.07 ± 0.97	−0.39 ± 1.30	0.09
V4–V1	0.61 ± 1.88	−1.04 ± 0.99^*∗∗*^	−1.24 ± 1.15^*∗∗*^	0.001
Secondary treatment				
V1	16.67 ± 5.31	17.52 ± 4.41	18.83 ± 3.28	0.41
V2	18.65 ± 6.02	19.04 ± 4.60	21.33 ± 3.63	0.28
V3	17.28 ± 5.83	16.30 ± 3.55	17.45 ± 3.54	0.76
V4	17.43 ± 5.86	16.09 ± 3.34	17.09 ± 3.50	0.70
V2-V1	1.98 ± 2.57	1.52 ± 0.92	2.50 ± 1.04	0.31
V3–V1	0.61 ± 1.91	−1.22 ± 1.13^*∗∗∗*^	−1.38 ± 0.86^*∗∗∗*^	<0.001
V4–V1	0.76 ± 1.50	−1.43 ± 1.35^*∗∗∗*^	−1.74 ± 0.54^*∗∗∗*^	<0.001
Third treatment				
V1	16.71 ± 5.24	16.67 ± 4.06	17.97 ± 3.26	0.65
V2	18.09 ± 5.44	18.34 ± 3.47	20.41 ± 3.34	0.27
V3	16.87 ± 5.07	15.83 ± 3.98	16.49 ± 3.04	0.79
V4	17.27 ± 5.27	15.47 ± 3.88	16.25 ± 3.11	0.51
V2-V1	1.38 ± 1.63	1.67 ± 1.54	2.45 ± 1.19	0.13
V3–V1	0.16 ± 1.08	−0.84 ± 0.37^*∗∗∗*^	−1.47 ± 0.73^*∗∗∗*^	<0.001
V4–V1	0.56 ± 2.07	−1.20 ± 0.38^*∗∗∗*^	−1.72 ± 0.61^*∗∗∗*^	<0.001
Fourth treatment				
V1	17.35 ± 4.79	16.04 ± 4.26	16.50 ± 2.97	0.68
V2	18.52 ± 5.53	17.77 ± 4.17	19.89 ± 3.47	0.44
V3	17.09 ± 5.33	15.24 ± 3.82	15.55 ± 3.25	0.45
V4	17.17 ± 4.86	15.06 ± 3.78	15.22 ± 3.02	0.28
V2-V1	1.17 ± 1.30	1.73 ± 1.10	3.39 ± 1.33^*∗∗∗###*^	<0.001
V3–V1	−0.26 ± 1.03	−0.81 ± 0.65	−0.95 ± 0.60	0.05
V4–V1	−0.17 ± 0.96	−0.98 ± 0.74^*∗∗*^	−1.28 ± 0.68^*∗∗*^	0.002

Data presented as mean ± standard deviation. SG, sham group; AG, acupuncture group; EG, electroacupuncture group; V1, first assessment; V2, second assessment; V3, third assessment; V4, fourth assessment; V2-V1, the intraocular pressure difference between V2 and V1; V3–V1, the intraocular pressure difference between V3 and V1; V4–V1, the intraocular pressure difference between V4 and V1. The intervention and group effect were measured by the repeated measures analysis of variance. ^*∗∗*^*p* < 0.01, ^*∗∗∗*^*p* < 0.001 compared to SG; ^###^*p* < 0.001 compared to AG.

**Table 4:** Effect of acupuncture on left eye intraocular pressure in glaucoma patients (mmHg).

	SG (*n* = 15)	AG (*n* = 14)	EG (*n* = 15)	*p* value
First intervention				
V1	16.88 ± 3.98	19.34 ± 3.91	20.66 ± 3.44^*∗*^	0.03
V2	18.10 ± 4.73	20.50 ± 4.40	22.27 ± 3.19	0.03
V3	16.65 ± 4.44	17.48 ± 3.61	19.56 ± 3.36	0.11
V4	17.01 ± 4.49	17.78 ± 3.59	19.00 ± 3.07	0.36
V2-V1	1.22 ± 1.50	1.16 ± 0.88	1.61 ± 0.67	0.49
V3–V1	−0.23 ± 1.07	−1.86 ± 1.61^*∗*^	−1.10 ± 1.45^*∗*^	0.01
V4–V1	0.13 ± 1.19	−1.56 ± 0.99^*∗∗*^	−1.66 ± 1.74^*∗∗*^	0.002
Second intervention				
V1	16.82 ± 3.64	17.91 ± 4.20	19.04 ± 3.21	0.27
V2	18.41 ± 4.74	19.04 ± 4.23	21.55 ± 3.00	0.11
V3	16.90 ± 4.39	16.36 ± 3.25	17.93 ± 3.54	0.52
V4	16.74 ± 4.07	16.29 ± 3.26	17.39 ± 3.23	0.70
V2-V1	1.59 ± 1.97	1.50 ± 0.93	2.51 ± 1.19	0.12
V3–V1	0.08 ± 1.55	−1.55 ± 1.29^*∗∗∗*^	−1.11 ± 1.05^*∗∗∗*^	<0.001
V4–V1	−0.08 ± 1.34	1.62 ± 1.21^*∗∗∗*^	−1.65 ± 0.75^*∗∗∗*^	<0.001
Third intervention				
V1	16.60 ± 4.37	17.03 ± 3.92	17.70 ± 3.28	0.74
V2	17.73 ± 4.34	18.57 ± 3.94	20.20 ± 3.64	0.24
V3	16.62 ± 4.15	15.99 ± 3.64	16.54 ± 2.94	0.88
V4	16.97 ± 4.16	15.83 ± 3.77	16.37 ± 3.06	0.71
V2-V1	1.13 ± 1.17	1.54 ± 0.65	2.50 ± 1.43^*∗∗∗*^	0.007
V3–V1	0.02 ± 1.19	−1.04 ± 0.66	−1.16 ± 0.81	<0.001
V4–V1	0.37 ± 1.48	−1.20 ± 0.64^*∗∗∗*^	−1.33 ± 0.71^*∗∗∗*^	<0.001
Fourth intervention				
V1	16.66 ± 3.63	16.41 ± 3.94	16.53 ± 2.96	0.98
V2	18.45 ± 3.94	17.96 ± 3.82	19.63 ± 3.16	0.46
V3	16.96 ± 3.28	15.53 ± 3.45	15.67 ± 3.10	0.43
V4	16.86 ± 3.32	15.19 ± 3.75	15.37 ± 3.05	0.34
V2-V1	1.79 ± 1.58	1.56 ± 0.98	3.10 ± 1.15^*∗∗*^	0.004
V3–V1	0.30 ± 0.96	−0.88 ± 0.88^*∗∗∗*^	−0.85 ± 0.63^*∗∗∗*^	<0.001
V4–V1	0.20 ± 0.63	−1.22 ± 0.88^*∗∗∗*^	−1.16 ± 0.61^*∗∗∗*^	<0.001

Data presented as mean ± standard deviation. SG, sham group; AG, acupuncture group; EG, electroacupuncture group; V1, first assessment; V2, second assessment; V3, third assessment; V4, fourth assessment; V2-V1, the intraocular pressure difference between V2 and V1; V3–V1, the intraocular pressure difference between V3 and V1; V4–V1, the intraocular pressure difference between V4 and V1. The intervention and group effect were measured by the repeated measures analysis of variance. ^*∗∗*^*p* < 0.01, ^*∗∗∗*^*p* < 0.001 compared to SG.

**Table 5:** The effect of acupuncture on the score of WHOQOL-BREF (Taiwanese version) in patients with glaucoma.

	SG (*n* = 15)	AG (*n* = 14)	EG (*n* = 15)	*p* value
Overall quality of life				
Overall evaluation of quality of life				
V1	3.47 ± 0.64	3.71 ± 0.73	3.67 ± 0.49	0.53
V2	3.67 ± 0.62	3.64 ± 0.50	3.67 ± 0.49	0.99
V2-V1	0.20 ± 0.41	−0.07 ± 0.47	0.00 ± 0.38	0.21
Overall health satisfaction				
V1	2.80 ± 0.56	2.86 ± 0.66	3.20 ± 0.86	0.26
V2	3.33 ± 0.62	3.21 ± 0.70	3.33 ± 0.82	0.88
V2-V1	0.53 ± 0.64	0.36 ± 0.50	0.13 ± 0.35	0.11
Physiological domain				
V1	14.40 ± 2.22	14.94 ± 1.57	15.35 ± 2.68	0.50
V2	14.86 ± 2.13	15.80 ± 1.11	15.77 ± 2.27	0.33
V2-V1	0.46 ± 0.99	0.86 ± 1.02	0.42 ± 0.88	0.41
Psychological domain				
V1	14 ± 1.89	14.71 ± 0.99	14.44 ± 1.98	0.52
V2	14.22 ± 2.3	15.05 ± 0.97	15.07 ± 1.44	0.31
V2-V1	0.22 ± 1.15	0.33 ± 0.63	0.62 ± 1.11	0.53
Social relationship				
V1	15.11 ± 1.79	15.62 ± 0.81	16.09 ± 1.28	0.16
V2	15.11 ± 1.57	15.90 ± 1.22	16.36 ± 1.18	0.05
V2-V1	0.00 ± 0.71	0.29 ± 1.4	0.27 ± 0.90	0.71
Social relationship domain (Tw)				
V1	14.60 ± 1.80	15.29 ± 0.99	15.87 ± 1.19	0.05
V2	14.80 ± 1.74	15.71 ± 1.20	16.07 ± 1.22	0.05
V2-V1	0.2 ± 0.68	0.43 ± 1.28	0.2 ± 0.56	0.73
Environment				
V1	14.27 ± 2.12	14.29 ± 1.79	14.63 ± 1.37	0.82
V2	14.53 ± 2.29	14.32 ± 2.17	14.90 ± 1.09	0.71
V2-V1	0.27 ± 0.82	0.04 ± 0.89	0.27 ± 0.78	0.69
Environment (Tw)				
V1	14.46 ±1.90	14.51 ± 1.75	14.70 ± 1.25	0.92
V2	14.70 ± 2.09	14.54 ± 2.01	14.99 ± 1.06	0.78
V2-V1	0.24 ± 0.75	0.03 ± 0.81	0.30 ± 0.66	0.61
Total scores of life qualities				
V1	99.33 ± 10.63	102.71 ± 7.63	104.33 ± 9.90	0.35
V2	101.93 ± 12.85	105.50 ± 7.67	107.00 ± 8.43	0.37
V2-V1	2.60 ± 4.31	2.79 ± 3.58	2.67 ± 3.11	0.99

Data presented as mean ± standard deviation. SG, sham group; AG, acupuncture group; EG, electroacupuncture group; WHOQOL-BREF (Taiwanese version), the World Health Organization Quality Of Life brief, Taiwanese version; V1, first assessment before the first intervention; V2, second assessment after finishing the fourth intervention; V2-V1, the score difference between V2 and V1; (Tw), Taiwanese version. The intervention and group effect were measured by the repeated measures analysis of variance.

## Data Availability

The data used to support the findings of this study are available from the corresponding author upon request.
